# Obinutuzumab as salvage anti-CD20 therapy in poly-refractory difficult-to-treat seropositive rheumatoid arthritis

**DOI:** 10.1093/rap/rkag089

**Published:** 2026-08-10

**Authors:** Irene Monjo-Henry, Marta Novella-Navarro, Marina Molinari, María Sanz-Jardón, M Gema Bonilla-Hernán, María Ángeles González-Fernández, Eugenio de Miguel Mendieta, Chamaida Plasencia-Rodríguez

**Affiliations:** Rheumatology Department, La Paz University Hospital, La Paz Institute for Health Research (IdiPAZ), Madrid, Spain; Rheumatology Department, La Paz University Hospital, La Paz Institute for Health Research (IdiPAZ), Madrid, Spain; Rheumatology Department, La Paz University Hospital, La Paz Institute for Health Research (IdiPAZ), Madrid, Spain; Rheumatology Department, La Paz University Hospital, La Paz Institute for Health Research (IdiPAZ), Madrid, Spain; Rheumatology Department, La Paz University Hospital, La Paz Institute for Health Research (IdiPAZ), Madrid, Spain; Pharmacy Department, La Paz University Hospital, La Paz Institute for Health Research (IdiPAZ), Madrid, Spain; Rheumatology Department, La Paz University Hospital, La Paz Institute for Health Research (IdiPAZ), Madrid, Spain; Rheumatology Department, La Paz University Hospital, La Paz Institute for Health Research (IdiPAZ), Madrid, Spain

Key message • Obinutuzumab may be an effective rescue anti-CD20 strategy in highly refractory seropositive D2T RA.

Sir, Difficult-to-treat rheumatoid arthritis (D2T RA) is defined by EULAR as persistent active or progressive disease despite recommended therapy, after failure of at least two biologic or targeted synthetic DMARDs with different mechanisms of action, often with ongoing symptoms, objective inflammation and/or inability to taper glucocorticoids [1]. This heterogeneous condition may reflect both inflammatory and non-inflammatory drivers, as well as comorbidities [2]. Clinical experience with obinutuzumab in RA remains scarce, although a recent case report described its safe and effective use in D2T RA after rituximab-induced allergy [3]. Here, we report a patient with long-standing seropositive erosive RA and high inflammatory burden in whom obinutuzumab achieved marked clinical improvement with parallel ultrasound-confirmed remission.

A woman was diagnosed with seropositive RA in May 2010, aged 36, after 12 months of inflammatory symptoms, fulfilling the 2010 ACR/EULAR classification criteria [4]. She smoked 2–3 cigarettes/day and had a grandmother with RA. Her course was marked by erosive disease, persistent biochemical and ultrasound inflammatory activity and chronic glucocorticoid exposure. In 2024, after developing xerophthalmia and xerostomia, she was diagnosed with associated Sjögren’s syndrome, fulfilling the 2016 ACR/EULAR classification criteria (a minor salivary gland biopsy focus score of 3.4 and bilateral Schirmer test of 4 mm), with ANA 1:2560 homogeneous pattern and negative anti-Ro/SSA and anti-La/SSB antibodies.

Initial treatment included prednisone 10 mg/day and methotrexate up to 25 mg/week. Intravenous tocilizumab was started in 2012 within an ongoing clinical trial and discontinued after 18 months because of secondary loss of efficacy, partly in the context of hypertransaminasemia. Certolizumab pegol (2013) showed primary non-response at 6 months (DAS28-ESR 4.70 to 5.16), and rituximab (2014) was stopped during the first cycle because of an infusion reaction. Abatacept (2014), etanercept (2016) and tofacitinib (2020) were discontinued because of secondary loss of efficacy, whereas combined etanercept plus tofacitinib (2021) achieved DAS28-ESR 2.51 at 6 months but was stopped after 18 months because of severe infection. Upadacitinib (2022) showed primary non-response. Rituximab was reintroduced in 2023 with desensitization but again showed primary non-response in the presence of anti-rituximab antibodies. Sarilumab (2023) showed primary non-response, followed by secondary loss of efficacy with baricitinib (2024) and infliximab plus methotrexate (2024) showed primary non-response despite add-on leflunomide 10 mg/day. Apart from the tocilizumab period complicated by hypertransaminasemia, all treatment lines included concomitant methotrexate. Detailed longitudinal DAS28-ESR, tender joint count and swollen joint count at treatment initiation and at discontinuation for each treatment line are provided in Supplementary Table S1 and Fig. S1, available at *Rheumatology Advances in Practice* Online.

Obinutuzumab was started in January 2025 as an off-label alternative anti-CD20 strategy, given persistent inflammatory activity and glucocorticoid dependence. At baseline, disease activity was high despite prednisone 15 mg/day (DAS28-ESR 7.1), with active synovitis on ultrasound (Fig. 1). DAS28-ESR improved only gradually during the first 6 months, with progressive glucocorticoid tapering and ultrasound improvement (Fig. 1). At 9 months, DAS28-ESR had fallen to 4.3 and prednisone was discontinued. By 12 months, DAS28-ESR was 2.5, the patient remained off glucocorticoids and ultrasound showed complete resolution of inflammatory activity (Fig. 1). No hypogammaglobulinemia was detected during follow-up (Supplementary Table S2, available at *Rheumatology Advances in Practice* Online). Detailed DAS28-ESR, tender joint count and swollen joint count evolution during obinutuzumab treatment is shown in Fig. 1A and B.

**Figure 1:**
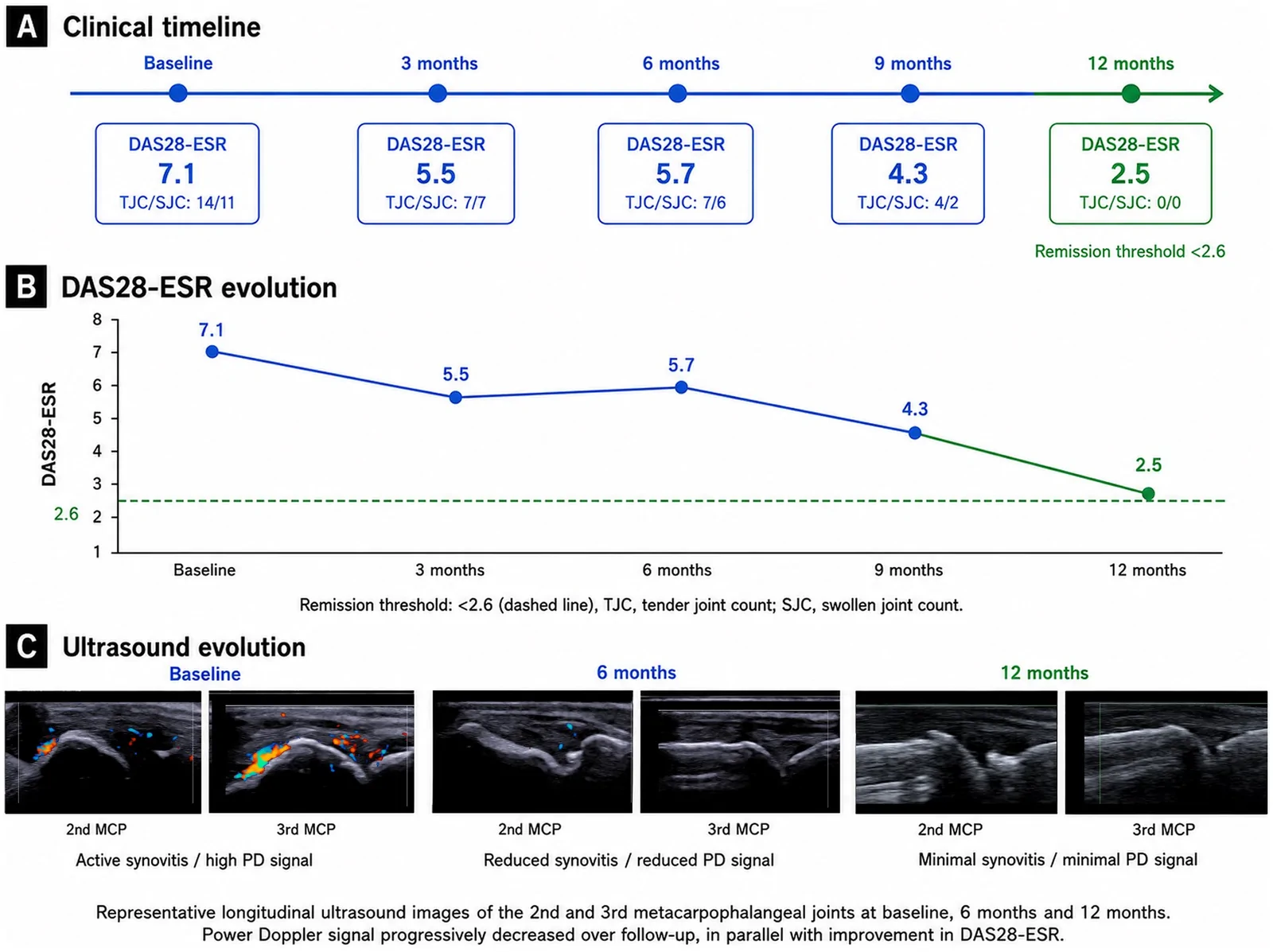
Longitudinal clinical and ultrasound response after obinutuzumab treatment. (A) Clinical timeline showing DAS28-ESR scores and tender/swollen joint counts at baseline and at 3, 6, 9 and 12 months of follow-up. Disease activity progressively improved from high activity at baseline, with DAS28-ESR decreasing from 7.1 to 2.5 at 12 months, reaching remission according to the DAS28-ESR threshold of <2.6. (B) Evolution of DAS28-ESR over time, illustrating sustained clinical improvement, particularly between months 6 and 12. The dashed green line indicates the remission threshold. (C) Representative longitudinal ultrasound images of the second and third metacarpophalangeal joints at baseline, 6 and 12 months. Baseline images showed active synovitis with marked Power Doppler signal. At 6 months, synovitis and Doppler activity were reduced, and by 12 months only minimal residual synovitis with absent Doppler signal was observed, paralleling the clinical improvement. Abbreviations: DAS28-ESR: Disease Activity Score in 28 joints using erythrocyte sedimentation rate; MCP: metacarpophalangeal joint; PD: Power Doppler; SJC: swollen joint count; TJC: tender joint count

This patient fulfilled the EULAR definition of D2T RA, with failure of multiple b/tsDMARDs across different mechanisms of action, persistent objective inflammation and glucocorticoid dependence [1]. Although associated Sjögren’s syndrome may have contributed to disease complexity, poorly controlled seropositive erosive RA preceded sicca onset by more than a decade, supporting refractory RA as the main clinical issue. A Sjögren’s-related effect on tender joint count cannot be excluded, but persistent xerophthalmia despite articular improvement and ultrasound-confirmed synovitis resolution support inflammatory RA control as the main driver of DAS28 improvement. Overall, this case is representative of inflammation-driven D2T RA and underscores the importance of reassessing patients to distinguish inflammatory from non-inflammatory contributors and identify factors underlying apparent DMARD failure [2, 5]. After failure of several TNF inhibitors and JAK inhibitors, further cycling within previously used therapeutic classes was considered unlikely to provide sustained benefit. Obinutuzumab is a glycoengineered type II anti-CD20 monoclonal antibody with enhanced effector function compared with rituximab. In RA and SLE samples, it has shown superior B cell cytotoxicity in whole-blood assays [6]. Therapeutic evidence in D2T RA remains limited, and management often relies on individualized, mechanism-based decisions [7]. In this highly refractory context, with previous rituximab hypersensitivity and anti-rituximab antibodies, obinutuzumab was selected as an individualized alternative anti-CD20 strategy. While the previously published RA case supports the potential safety and effectiveness of obinutuzumab after rituximab-induced allergy [3], our report adds longitudinal ultrasound-confirmed remission, detailed DAS28 component data and serial immunoglobulin monitoring after rituximab desensitization failure with anti-rituximab antibodies. In summary, this case suggests that obinutuzumab may represent a valuable rescue option in highly refractory seropositive RA, with clinically meaningful improvement, successful glucocorticoid withdrawal and objective imaging-confirmed remission, even after rituximab hypersensitivity and anti-rituximab antibodies.

## Supplementary Material

rkag089_Supplementary_Data

## Data Availability

The data underlying this article will be shared on reasonable request to the corresponding author.
