# Correction: Obinutuzumab as salvage anti-CD20 therapy in poly-refractory difficult-to-treat seropositive rheumatoid arthritis

**DOI:** 10.1093/rap/rkag106

**Published:** 2026-10-15

This is a correction to: Irene Monjo-Henry, Marta Novella-Navarro, Marina Molinari, María Sanz-Jardón, M Gema Bonilla-Hernán, María Ángeles González-Fernández, Eugenio de Miguel Mendieta, Chamaida Plasencia-Rodríguez, Obinutuzumab as salvage anti-CD20 therapy in poly-refractory difficult-to-treat seropositive rheumatoid arthritis, *Rheumatology Advances in Practice*, Volume 10, Issue 3, 2026, rkag089, https://doi.org/10.1093/rap/rkag089

The following change has been made to the originally published paper.

Text in the first sentence of the second paragraph is corrected to read: “A woman was diagnosed with seropositive RA in May 2010, aged 36,[…]” instead of: “A woman was diagnosed with seropositive RA in May 2010, aged 46,[…]”.

