# Supplementary material for: Obinutuzumab as salvage anti-CD20 therapy in poly-refractory difficult-to-treat seropositive rheumatoid arthritis
Source: Rheumatol Adv Pract. 2026 Aug 10;10(3):rkag089. doi: 10.1093/rap/rkag089 (PMC13461643; doi:10.1093/rap/rkag089)
Supplement: Supplementary file 1 [file rkag089_supplementary_data.docx]

**Supplementary Table S1.** Clinical evolution across treatment lines

| **#** | **Treatment** | **Start year** | **Baseline DAS28-ESR** | **Baseline TJC** | **Baseline SJC** | **Final DAS28-ESR** | **Final TJC** | **Final SJC** | **Outcome / reason for discontinuation** |
| --- | --- | --- | --- | --- | --- | --- | --- | --- | --- |
| 1 | Tocilizumab IV | 2012 | 5.17 | 6 | 5 | 4.17 | 6 | 4 | Secondary loss of efficacy; discontinued in the context of hypertransaminasaemia |
| 2 | Certolizumab pegol | 2013 | 4.70 | 6 | 2 | 5.16 | 10 | 4 | Primary non-response |
| 3 | Rituximab | 2014 | 4.20 | 6 | 3 | N/A^a^ | N/A^a^ | N/A^a^ | Discontinued during first infusion cycle due to infusion reaction |
| 4 | Abatacept | 2014 | 5.70 | 7 | 5 | 5.13 | 5 | 3 | Secondary loss of efficacy |
| 5 | Etanercept | 2016 | 5.86 | 6 | 5 | 6.12 | 12 | 12 | Secondary loss of efficacy |
| 6 | Tofacitinib | 2020 | 6.80 | 13 | 8 | 5.93 | 9 | 10 | Secondary loss of efficacy |
| 7 | Etanercept + tofacitinib | 2021 | 6.06 | 11 | 10 | 4.96 | 4 | 4 | Discontinued due to severe infection |
| 8 | Upadacitinib | 2022 | 6.86 | 16 | 11 | 5.86 | 9 | 6 | Primary non-response |
| 9 | Rituximab with desensitisation | 2023 | 5.13 | 10 | 9 | 4.65 | 8 | 6 | Primary non-response in the presence of anti-rituximab antibodies |
| 10 | Sarilumab | 2023 | 7.30 | 20 | 19 | 5.19 | 13 | 11 | Primary non-response |
| 11 | Baricitinib | 2024 | 5.10 | 13 | 11 | 5.02 | 5 | 6 | Secondary loss of efficacy |
| 12 | Infliximab | 2024 | 6.99 | 19 | 11 | 7.20 | 11 | 12 | Primary non-response |
| 13 | **Obinutuzumab** | January 2025 | 7.10 | 14 | 11 | **2.50** | **0** | **0** | Marked clinical improvement |

^a^ Not applicable: treatment was discontinued during the first infusion cycle due to infusion reaction; post-treatment assessment was not performed. DAS28-ESR, Disease Activity Score in 28 joints using erythrocyte sedimentation rate; SJC, swollen joint count; TJC, tender joint count. Row 13 (obinutuzumab, shaded green) denotes the first and only treatment to achieve sustained clinical remission (DAS28-ESR <2.6), with glucocorticoid withdrawal and ultrasound remission at 12 months. Treatment is ongoing at the time of writing.

**Supplementary Figure S1.** Baseline and final disease activity across treatment lines. Baseline and final values, defined as the last available assessment before treatment switch or discontinuation, are shown for each treatment line for DAS28-ESR (S1A), tender joint count (TJC; S1B), and swollen joint count (SJC; S1C). Blue bars represent baseline values and orange bars represent final values. In S1A, the dashed green line indicates the DAS28-ESR remission threshold (<2.6), with values above this threshold reflecting active disease. Obinutuzumab was the only treatment associated with a final DAS28-ESR below the remission threshold (2.50 at 12 months), together with final TJC and SJC values of 0. The first rituximab exposure was not evaluable for final disease activity because treatment was discontinued during the first infusion cycle due to an infusion reaction.

**Abbreviations**

ABA, abatacept; BARI, baricitinib; CZP, certolizumab pegol; DAS28-ESR, Disease Activity Score in 28 joints using erythrocyte sedimentation rate; ETA, etanercept; ETA+TOFA, etanercept plus tofacitinib combination; IFX, infliximab; OBI, obinutuzumab; RTX, rituximab (first cycle, interrupted due to infusion reaction); RTX+des., rituximab with desensitisation protocol; SAR, sarilumab; SJC, swollen joint count; TCZ-IV, tocilizumab intravenous; TJC, tender joint count; TOFA, tofacitinib; UPA, upadacitinib.

**Supplementary Table S2**. Evolution of immunoglobulin levels during treatment with obinutuzumab

| **Time point** | **IgG (mg/dL)** | **IgM (mg/dL)** | **IgA (mg/dL)** |
| --- | --- | --- | --- |
| Baseline | 1366 | 356 | 375 |
| 3 months | 1021 | 161 | 251 |
| 6 months | 977 | 118 | 230 |
| 9 months | 959 | 111 | 214 |
| 12 months | 946 | 101 | 213 |

Ig, immunoglobulin. No hypogammaglobulinemia or infectious complications were observed during follow-up.
